# Outpatient versus inpatient superficial parotidectomy: clinical and pathological characteristics

**DOI:** 10.1186/s40463-020-00484-9

**Published:** 2021-02-12

**Authors:** Daniel J. Lee, David Forner, Christopher End, Christopher M. K. L. Yao, Shireen Samargandy, Eric Monteiro, Ian J. Witterick, Jeremy L. Freeman

**Affiliations:** 1 Department of Otolaryngology – Head & Neck Surgery, University of Toronto, Toronto, ON Canada; 2 Division of Otolaryngology – Head & Neck Surgery, Department of Surgery, Dalhousie University, Halifax, ON Canada; 3 Faculty of Medicine, University of Toronto, Toronto, ON Canada; 4 Department of Otolaryngology – Head & Neck Surgery, Sinai Health System, 600 University Avenue, Suite 401, Toronto, ON M5G 1X5 Canada

**Keywords:** Parotidectomy, Outpatient surgery, Ambulatory surgery

## Abstract

**Background:**

Superficial parotidectomy has a potential to be performed as an outpatient procedure. The objective of the study is to evaluate the safety and selection profile of outpatient superficial parotidectomy compared to inpatient parotidectomy.

**Methods:**

A retrospective review of individuals who underwent superficial parotidectomy between 2006 and 2016 at a tertiary care center was conducted. Primary outcomes included surgical complications, including transient/permanent facial nerve palsy, wound infection, hematoma, seroma, and fistula formation, as well as medical complications in the postoperative period. Secondary outcome measures included unplanned emergency room visits and readmissions within 30 days of operation due to postoperative complications.

**Results:**

There were 238 patients included (124 in outpatient and 114 in inpatient group). There was no significant difference between the groups in terms of gender, co-morbidities, tumor pathology or tumor size. There was a trend towards longer distance to the hospital from home address (111 Km in inpatient vs. 27 in outpatient, mean difference 83 km [95% CI,- 1 to 162 km], *p* = 0.053). The overall complication rates were comparable between the groups (24.2% in outpatient group vs. 21.1% in inpatient, *p* = 0.56). There was no difference in the rate of return to the emergency department (3.5% vs 5.6%, *p* = 0.433) or readmission within 30 days (0.9% vs 0.8%, *p* = 0.952).

**Conclusion:**

Superficial parotidectomy can be performed safely as an outpatient procedure without elevated risk of complications.

**Graphical abstract:**

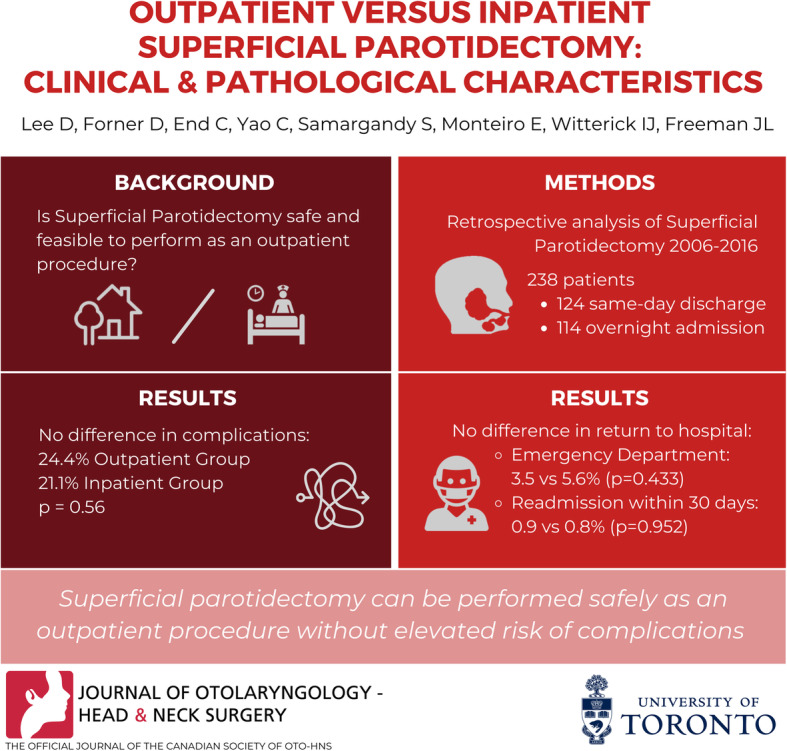

## Introduction

Outpatient surgeries have gained popularity in modern healthcare with potential for improving overall efficiency in healthcare resource utilization [1–3]. This trend has been accompanied by advances in anesthetic and surgical technology, allowing for safe perioperative patient care on an outpatient basis [1–3]. In fact, many “low” to “moderate” risk surgical procedures such as arthroscopy, cholecystectomy and hernia repair are routinely performed in ambulatory surgical centres [1, 2, 4–6]. In the realm of head and neck surgery, thyroidectomy has emerged as a viable candidate for outpatient surgery with an acceptable safety profile [7]. Common criteria for determining candidacy for outpatient procedures include lack of significant co-morbidities, close proximity to a hospital, and availability of adequate social support [7, 8].

Parotidectomy is performed to treat primary and malignant tumors and to manage non-neoplastic conditions, such as chronic sialadenitis. In the modern era, it can be classified as extracapsular dissection, superficial or total parotidectomy based on the extent of resection. Although historically considered to be an inpatient procedure, this decision may be largely borne out of practice norms rather than evidence-based medicine. In fact, the feasibility of ambulatory parotidectomy has been demonstrated in a number of retrospective reports [9–15]. However, some of these studies were limited by small sample sizes [10, 12, 15] while others had heterogeneous comparison groups with different extent and duration of the operation [11, 13, 14]. Therefore, the aim of this study was to evaluate the safety profile and feasibility of outpatient superficial parotidectomy compared to inpatient superficial parotidectomy at a tertiary academic centre.

## Methods

### Study design and patient population

Research approval was granted by the Research Ethics Board at Sinai Health System. A retrospective review of adult patients who underwent superficial parotidectomy with preservation of the facial nerve at Mount Sinai Hospital in Toronto, Ontario between 2006 and 2016 was performed. Patients were identified from an institutional pathology database. The extent of the surgery was determined from the operative note. Patients were excluded if they had total parotidectomy, resection of a deep lobe tumor, free flap reconstruction, history of radiation or previous resection at the surgical site, pre-operative diagnosis of malignant tumors, or incomplete documentation. Malignancy was excluded due to the anticipated extent of resection required and the necessity of inpatient monitoring. We included those who underwent limited neck dissection for lymph node biopsy at the time of parotidectomy, and these procedures were evenly performed in the inpatient and outpatient groups (42.1% vs 42.7%, *p* = 0.921).

### Definitions: “outpatient” vs. “inpatient”

For the purpose of this investigation, two groups were created based on the length of stay. The outpatient group consisted of patients who were discharged home the same day from the post-anesthesia care unit after the surgery. The inpatient group included those who stayed at least one night following the surgery. At our institution, there is no predefined criteria for performing outpatient vs inpatient parotidectomy, and therefore the decision for an outpatient procedure was at the discretion of the attending surgeon. In general, patients selected for outpatient parotidectomy are free from significant comorbidities, have adequate home assistance, are reliable in their own care, and live relatively close to hospital. Length of follow-up was defined as the time between the date of surgery and the last visit Otolaryngology clinic visit. Drains were placed at the discretion of the attending surgeons and removed if the drain output was less than 30 cc in 24 h. Patients were discharged home with home care if they underwent ambulatory surgery or if the drain output was more than the pre-defined output during the inpatient stay.

### Outcome measurements and data collection

The data were extracted from electronic and scanned medical chart reviews reviews using a standardized data extraction form by CE and verified by DJL and DF. Discrepancies were reviewed and resolved by the senior author. Demographic characteristics were collected and included age, gender and geographic proximity to the hospital. Geographic proximity was calculated using Google Maps (Google LLC) and the patient’s most recent postal code on record. The driving distance and travel time were recorded based on the minimum amount of time estimated by Google Maps travelling from Mount Sinai Hospital to the patient’s address at 10:00 am the same day as their operation to control for anticipated traffic variations. Medical co-morbidities at the time of surgery were recorded from chart reviews including consultation notes and anesthesia records. We utilized the Charlson Co-morbidity Index (CCI) to score the noted medical co-morbidities [16, 17]. Inpatient records, including operative notes, were reviewed for surgical indication, drain management, and length of in-hospital stay. Pathology reports were reviewed for maximum dimension, volume (length x width x height as provided in the pathology report) and the pathological diagnosis of the tumor. Measurements are representative of a combination of tumor and specimen sizes.

The primary outcome was the number of postoperative complications, including hematoma, seroma, transient/permanent facial nerve palsy, wound infection and fistula formation as well as any medical complications in the immediate postoperative period. Secondary outcome measures included the number of emergency room visits and unplanned admissions within first 30 days of operation due to postoperative complications.

### Statistical analysis

All statistical analysis and database maintenance was performed using SPSS Statistics (IBM SPSS Statistics for Windows, version 21 (IBM Corp., Armonk, N.Y., USA) and SAS Studio (SAS® Institute, Cary NC). Central tendency of nominal and ordinal data was described as median frequencies. Central tendency of parametric data was presented as means, while that of non-parametric interval and ratio data was presented as medians.

Categorical data was compared using Pearson’s Chi-squared (χ^2^) test or Fisher’s exact test. The CCI ordinal variable was analyzed using Mann-Whitney U-Test. Continuous variables were compared using independent sample two-tailed Student’s T-Test for parametric data and Mann-Whitney U-Test for non-parametric data. Statistical significance thresholds (alpha) were a priori set to 0.05 and 95% confidence intervals were used for continuous variables.

## Results

### Study population and baseline characteristics

A total of 238 patients fit the inclusion criteria. Twenty-two patients with malignant tumors were excluded. There were 124 patients in the outpatient group and 114 patients in the inpatient group. Baseline characteristics are shown in Table 1. At baseline, the inpatient group was statistically older than the outpatient group but there was no difference in gender distribution. In addition, the co-morbidity burden was no different between the groups (CCI median 1 [IQR = 2 in outpatient vs. 1 [IQR = 3] in inpatient, *p* = 0.169]. Patients in the inpatient group had significantly longer driving time from home to hospital (more than 60 min) compared to those in the outpatient group (*p* < 0.001). There was a trend towards longer distance to the hospital from home address (111 Km in inpatient vs. 27 in outpatient, mean difference 83 km [95% CI,- 1 to 162 km], *p* = 0.053). In the inpatient group, the median length of stay was 1 day (range 1–7 days). The majority of patients stayed only 1 day (70.2%) or 2 days (24.6%), with only a few patients requiring 3 days of stay (1.8%), 4 days (2.6%), or 7 days (0.9%).

**Table 1:** Patient Demographics

	Inpatient	Outpatient	statistic
Age (mean (range))	54 (16–85)	50 (19–85)	***p*** **= 0.040**
Male Gender (n (%))	53 (46.5)	49 (39.5)	*p* = 0.277
Distance (km (range))	115.9 (1.0–152.0)	26.9 (1.4–1984)	p = 0.053
Travel Time			**p < 0.001**
< 30 min	52 (45.6)	54 (43.5)	
30–60 min	45 (39.5)	68 (54.8)	
> 60 min	17 (14.9)	2 (1.6)	
CCI (median score (IQR))	1 (3)	1 (3)	
Charlson distribution (n (%))			p = 0.169
0	38 (33.3)	56 (45.2)	
1	24 (21.1)	28 (22.6)	
2	22 (19.3)	23 (18.5)	
3	16 (14.0)	7 (5.6)	
4	10 (8.8)	7 (5.6)	
5	4 (3.5)	2 (1.6)	
6	0 (0.)	1 (0.8)	

*p-value significance threshold set at ≤0.05

Age (years), distance (kilometers), *CCI* Charlson comorbidity index

### Surgical and pathological characteristics

Two-hundred six (86.6%) patients had information available regarding fine needle aspirate (FNA) biopsy. The most common pre-operative cytological diagnosis from FNA was pleomorphic adenoma (52.4%), followed by Warthin’s tumor (13.1%), negative for malignancy (7.3%), and insufficient for diagnosis (6.8%). The remaining diagnoses included cellular atypia, benign, cystic, salivary gland neoplasm, lymphoid infiltrate, atypical cells, monomorphic adenoma, suspicious for malignancy, and reactive/inflammatory.

Superficial parotidectomies were performed in the standard fashion with a modified Blair incision, identification of the facial nerve, and extirpation of the tumor. Cases were performed by seven surgeons in total, of which three surgeons performed outpatient parotidectomies and six performed inpatient parotidectomies. One surgeon performed the majority of outpatient parotidectomies (84%), while inpatient parotidectomies were approximately equal across surgeons. Extracapsular dissections were not performed in this patient group. Overall, surgical drain placement was common practice. Surgical drains were placed significantly more often in the inpatient group than the outpatient group (97.4% vs 88.97%, *p* = 0.010). The was no difference in specimen size between the inpatient and outpatient groups, as determined by maximum dimension (median 27 mm vs. 25 mm, *p* = 0.203). Specimen or tumor volume was available for 220 patients (92%, 109 outpatients and 111 inpatients). There was no difference in volume between the inpatient and outpatient group (median 80.0 mm^3^ versus 65.3 mm^3^, *p* = 0.539). Pathological diagnoses were similar between the groups, with pleomorphic adenoma accounting for more than half of all diagnoses (*p* = 0.143, Table 2).

**Table 2:** Parotid Tumor pathology

Diagnosis (n (%))	Inpatient	Outpatient
Pleomorphic Adenoma	61 (53.5)	80 (64.5)
Warthin’s Tumor	29 (25.4)	19 (15.3)
Benign Lymphoepithelial cyst	3 (2.6)	1 (0.8)
Other pathology^a^	21 (18.4)	24 (19.4)

^a^Other pathology includes a wide range of low count diagnoses, including sialadenitis, basal cell adenoma, cystic lesions, myoepithelioma, neurofibroma, oncocytic cystadenoma, etc

### Postoperative outcomes

Mean length of follow-up overall was 26 months (range 0.07–150 months), which did not differ between the inpatient and outpatient groups (23.9 vs 27.6 months, *p* = 0.422). The overall parotid-specific complication rates were similar between the outpatient and inpatient groups (24.2% vs. 21.1%, *p* = 0.563) (Table 3). The most common complication was transient facial nerve weakness (13.7% in outpatient vs. 7.0% in inpatient), but this finding was not statistically significant (*p* = 0.093). There were no patients with permanent facial nerve paralysis in the outpatient group, while three (2.6%) patients in the inpatient group experienced permanent paralysis. Hematoma formation was more frequent in the inpatient group compared to the outpatient group (4.4% vs. 0%, *p* = 0.018). Of the 5 patients experiencing hematoma, 3 patients returned to the operating room for drainage, 1 patient underwent incision and drainage at the bedside and 1 remaining patient was conservatively managed with observation. There was no difference in seroma formation, wound infection, or development of Frey Syndrome between the groups. Concurrent limited neck dissection was not associated with increased rates of seroma (neck dissection 0.8% vs no dissection 0.4%, *p* = 0.748) or hematoma (1.7% vs 0.4%, *p* = 0.305. No patients reported fistulization. Only four non-parotidectomy specific complications occurred, all of which were in the inpatient group and were considered minor (transient ST depression, asymptomatic bradycardia, and two patients with post-operative nausea and vomiting). There was no significant difference between the inpatient and outpatient groups in return to the emergency department within 30 days (3.5% vs 5.6%, *p* = 0.433) or readmission within 30 days (0.9% vs 0.8%, *p* = 0.952). Eleven patients presented to the emergency department, predominately (5, (45.5%)) for non-specified postoperative complications and were discharged the same day. Otherwise, patients presented to the emergency department for postoperative pain (2, (18.2%)), swelling (3, (27.3%)), and cholelithiasis (1, (9.1%)). Of the two patients who were readmitted, one was for facial swelling, and the other for facial cellulitis.

**Table 3:** Complications

Complication	Inpatient	Outpatient	Statistic
Any complication (n, (%))	24 (21.1)	30 (24.2)	*p* = 0.563
Hematoma (n, (%))	5 (4.4)	0 (0)	***p*** **= 0.018**
Seroma (n, (%))	2 (1.8)	1 (0.8)	*p* = 0.513
Temporary facial nerve paresis (n, (%))	8 (7.0)	17 (13.7)	*p* = 0.093
Permanent facial nerve paralysis (n, (%))	3 (2.6)	0 (0)	*p* = 0.069
Wound infection (n, (%))	0 (0)	2 (1.6)	*p* = 0.173
Fistula (n, (%))	0 (0)	0 (0)	N/A
Frey’s Syndrome (n, (%))	4 (3.5)	10 (8.1)	*p* = 0.136
30-day ED visit (n, (%))	4 (3.5)	7 (5.6)	*p* = 0.433
30-day Readmission (n, (%))	1 (0.9)	1 (0.8)	*p* = 0.952

*p-value significance threshold set at ≤0.05

*ED* Emergency department

ED visit and Readmission are within 30 days of parotidectomy

## Discussion

Recently, ambulatory surgery has become more common across various surgical specialties as a means to provide quality care in our cost-conscious healthcare environment [1, 2, 5, 7, 18]. There is increasing interest in outpatient surgeries amongst otolaryngologists and head and neck surgeons. This has been reflected in uncomplicated head and neck surgeries, such as thyroidectomy and superficial parotidectomy.

Previous retrospective studies attempted to demonstrate the safety of outpatient parotidectomy in comparison to the inpatient procedure. However, in depth literature review reveals significant heterogeneity in the inpatient groups as many of them had deeper tumor size, included a greater proportion of total parotidectomy (up to 3 times more), and longer operative times (up to 25% longer) [11, 13, 14]. Given these, selection bias may have resulted in elevated risks of complications in the inpatient group, thereby assuming the safety of outpatient parotidectomy. Recent systematic reviews and meta-analyses also support the safety of outpatient parotidectomy, compared to inpatient parotidectomy, with comparable rates of complications and re-admissions [19, 20]. However, similar to our literature review findings, results of the systematic reviews are limited by the low quality of included studies especially in terms of comparability between outpatient and inpatient groups. Further, substantial heterogeneity exists amongst studies, both clinically and in the design of included studies [20]. Therefore, distinct from other studies, this retrospective cohort study purposefully included only those who had undergone superficial parotidectomy for non-malignant conditions, in an effort to reduce selection bias stemming from increased operative time with a more extensive operation. The results of our study provide further evidence in establishing a safety profile of outpatient superficial parotidectomy compared with inpatient superficial parotidectomy and provides additional clarity beyond that provided by previous evidence synthesis efforts.

In our series, there was no clinically significant difference in age between the groups, and there was no difference in gender or co-morbidities, which were collected using the standardized Charlson comorbidity index. In addition, the tumor pathology was similar along with the tumor sizes between the two groups. With the well-balanced demographics, co-morbidities and pathology diagnoses, the results of our study support the notion that the overall complication rates, emergency room visits and re-admission rates are similar between the outpatient and inpatient groups. Notably, hematoma was less frequent in the outpatient group and there was a trend toward increased rates of permanent facial nerve paralysis in the inpatient group. However, with the limits of retrospective study, it is difficult to explain this phenomenon, as the co-morbidities and defect sizes were similar between the groups. Further studies may investigate etiologies for these differences in outcomes, including extent of dissection, monitoring practices, and others. Regardless, the results show that superficial parotidectomy can be performed safely as an ambulatory procedure.

One of the limitations of ambulatory surgery is the lack of direct patient observation and management of pertinent postoperative issues in-hospital. One such issue is drain management. The current study demonstrates that surgical drain placement can be managed safely as an outpatient, as a substantial proportion of both groups received drains in our study with no differences in the rates of seroma or wound infections. Another consideration in performing outpatient surgery is proximity to a skilled facility in case of emergencies that require interventions. Appropriately, in our series, there was a shorter commute time in the outpatient group compared to the inpatient group, with the majority of patients living within 60 min of driving distance. Patients admitted to the hospital also tended to be older than those managed as an outpatient. Age, drain management and travel distance along with comorbidity burden are important considerations that should be included in discussion of outpatient procedure with patients.

Our study has a number of limitations that should be addressed. The retrospective nature exposes the study to biases, such as information bias. There is also the lack of a standardized pre-established protocol for determining candidacy for ambulatory surgery. At our institution, not all surgeons perform outpatient parotidectomy and this decision is largely personal preference. Indeed, despite no differences in comorbidities between those undergoing inpatient and outpatient parotidectomy, some surgeons did not perform outpatient surgery. This may be a result of continuing pre-established practice norms. These differences are also reflected in the rate of drain placement between the outpatient and inpatient groups, although the complication rates are comparable between the two. This may have contributed to potential selection bias in a retrospective review. However, these limitations were also present in the previous retrospective reviews on this topic. As well, this study does not delineate patients who were scheduled for outpatient surgery but were admitted post-operatively for anesthesia and recovery concerns, or for late operating room finishing time. Future prospective studies may help address this question. In addition, we do not provide patient-oriented outcomes, such as patient preferences or quality of life. A well-designed qualitative study in the future would be useful in determining patient centered outcomes for outpatient parotidectomy.

## Conclusion

This large series demonstrates that outpatient superficial parotidectomy can be performed safely with no increased risk of postoperative complications, emergency room visits or re-admissions.

## Data Availability

The datasets used and/or analyzed during the current study are available from the corresponding author on reasonable request.
